# Do adverse childhood experiences increase the risk of postdeployment posttraumatic stress disorder in US Marines?

**DOI:** 10.1186/1471-2458-10-437

**Published:** 2010-07-26

**Authors:** Cynthia A LeardMann, Besa Smith, Margaret AK Ryan

**Affiliations:** 1 Department of Defense Center for Deployment Health Research, Naval Health Research Center, San Diego, CA, USA

## Abstract

**Background:**

Posttraumatic stress disorder (PTSD) has been associated with combat intensity, lack of social support, and adverse childhood factors among military personnel in previous studies. It has not been well established if adverse childhood experiences reported predeployment are independently associated with postdeployment PTSD.

**Methods:**

Data were evaluated from 8,391 male responders of the Recruit Assessment Program survey at Marine Corps Recruit Depot in San Diego who were deployed in support of military conflicts between September 2001 and June 2004. Using patient medical records to determine PTSD diagnoses, Cox proportional hazard modeling was performed to examine if adverse childhood experiences were independently associated with postdeployment PTSD.

**Results:**

After adjustment, those who reported adverse childhood experiences in more than one category were significantly more likely to be diagnosed with postdeployment PTSD. Specifically, childhood physical neglect was mostly strongly associated with postdeployment PTSD.

**Conclusions:**

Findings suggest that Marines who experience multiple types of adverse childhood experiences may be at increased risk for postdeployment PTSD. It is possible, however, that these results indicate that men willing to report childhood adverse experiences are also more willing to seek care for PTSD.

## Background

Posttraumatic stress disorder (PTSD) can occur after a traumatic event, such as combat exposure, serious accident, and violent personal assault. However, not all individuals who survive a severely traumatic event develop PTSD. Certain factors may cause some people to be more vulnerable to developing PTSD than others [1]. It has been suggested that the psychological process that occurs during and immediately following a traumatic event affects an individual's post-event mental health status [2]. Specifically, those who respond to a traumatic event with intensely negative emotions, have dissociative experiences, and lack coping skills are at increased risk for PTSD. Factors, such as family history of mental illness and prior traumatic events, may lead to psychological difficulties, emotional dysregulation, and self-destructive behaviors [2,3]. In addition, individuals who have been maltreated as children are less likely to have well formed social networks for support [4]. Therefore individuals who have experienced childhood trauma may be more vulnerable to develop PTSD, as they have less ability to positively respond and cope with new traumatic events.

Among military members, intensity of combat exposure has consistently been demonstrated as one of the strongest predictors of subsequent combat-related PTSD [5-10], while many pretrauma and posttrauma factors have shown less-consistent associations with PTSD [7,8,11,12]. Adverse childhood experiences (ACE), including sexual, physical, emotional abuse; physical and emotional neglect; and family dysfunction, have also been well documented as risk factors for PTSD among military service members. In a meta-analysis of risk factors for PTSD, trauma severity, lack of social support, and adverse childhood factors (excluding abuse) were among the strongest predictors for PTSD among military service members [8]. Another study of 221 female veterans who visited the Veterans Affairs Healthcare System between 1998 and 1999 reported that childhood trauma, including emotional abuse, physical abuse, sexual abuse, emotional neglect, and physical neglect, contributed to the severity of PTSD [13]. Lapp [14] found that 69% of male veterans with a primary admitting diagnosis of PTSD had self-reported suffering either sexual or physical abuse as a child. Similarly, higher rates of childhood physical abuse were reported in patients with PTSD in a study of Vietnam combat veterans [15]. More recently, a study reported an association between ACE and the expression of mental illness among adults [16]. Among male US soldiers, ACE have been associated with an increase of mental health outcomes [17]. Also, an association was found between childhood adversity and PTSD symptoms among UK military personnel who had been deployed to Iraq [18].

The Recruit Assessment Program (RAP), launched June 2001 at the Marine Corps Recruit Depot (MCRD) in San Diego, California, was designed to collect baseline health data on Marine recruits. The RAP survey is administered during the first few days of recruit training. The recruits are informed that the survey is voluntary and their responses will not disqualify them from military service. The main goal of RAP is to collect and examine health and behavioral data at service entrance to better understand service-related exposures and health outcomes [19].

An association between ACE and mental health outcomes has been documented, however most previous studies have assessed ACE after the traumatic event, introducing potential biases related to recall and reporting [13-16,18]. The objective of this study was to prospectively examine the association between ACE and PTSD among Marines following deployment in support of military conflicts in Iraq and Afghanistan.

## Methods

### Population and Data Sources

As of March 2010, over 130,000 recruits had completed a RAP survey during initial training, with a response rate over 95%. Development of the RAP survey was a collaborative effort of public health officials, clinicians, and researchers from the Department of Defense, Veterans Health Administration, and Department of Health and Human Services. The survey instrument includes questions on demographics, health, family history, tobacco and alcohol use, mental health history, and ACE [19].

Adverse childhood experience questions on the RAP survey were derived from the Adverse Childhood Experiences Study [20,21], which adapted questions from the Childhood Trauma Questionnaire [22], Conflict Tactics Scales [23], and sexual abuse questions from Wyatt [24]. Using questions framed to reflect experiences prior to age 17 years, 7 ACE categories were assessed in the current study: (1) physical neglect (lack of care and protection), (2) emotional neglect (lack of feeling loved), (3) physical abuse (pushed, grabbed, shoved, or slapped), (4) emotional abuse (was sworn at, insulted, or put down), (5) sexual abuse, (6) domestic violence, and (7) family history of mental illness or alcohol abuse. Each ACE category was based on 1 self-reported question from the RAP survey, with the exception of family history of mental illness and alcohol abuse, which was assessed using 2 questions. The 7 ACE categories were evaluated as dichotomous "yes" or "no" responses. Questions with a 5-point scale were dichotomized based on criteria used by Dube et al. [21]; for neglect, a positive response was classified if responders reported neglect "often" or more frequently. A 3-level categorical variable was created to evaluate overall occurrences of childhood adversity: no history of ACE, history of ACE in 1 category, or history of ACE in 2 or more categories.

Other questions from the RAP survey were used as covariates in multivariable modeling, including education, parental education, number of close friends, alcohol-drinking behavior, smoking status, self-reported mental health status, visiting a mental health professional in the 5 years prior to survey completion, and prior traumatic event (including being in a severe accident, seeing someone badly injured or killed, being attacked or assaulted, threatened with a weapon, or being raped).

Deployment data, obtained from the Defense Manpower Data Center (DMDC), include all service members deployed in support of the conflicts in Iraq and Afghanistan since September 2001. Length of deployment was defined as the cumulative number of days deployed after completion of the RAP survey until first mental health event, 2 years after last deployment, or June 30, 2005, whichever came first. Marital status, age at first deployment, Armed Forces Qualification Test (AFQT) score, and race/ethnicity were also acquired from DMDC. The AFQT score is computed using four tests that measure knowledge in high school level subjects.

Patient medical records were used to determine diagnosis for PTSD, and mental health disorders that may be manifestations of PTSD. Hospitalization and outpatient data sources, including all visits to military treatment facilities as well as civilian visits billed to the military, were used in these analyses. All diagnostic fields were scanned for PTSD (*ICD-9-CM *codes 308 and 309.8) and other mental health disorders (*ICD-9-CM *codes 290, 293-298, 300, 301, 306, 308, 309-312, and 316), excluding disorders specific to childhood, sexual deviations, special syndromes, mental retardation, and substance abuse or dependence. Coding was based on the *International Classification of Diseases, Ninth Revision, Clinical Modification *(*ICD-9-CM*) [25]. For the purpose of this investigation, responders were classified as having an outcome if one of the *ICD-9-CM*-coded diagnoses was listed in the medical record within 24 months following deployment and prior to the end of the study period. Diagnoses during deployment often do not appear in electronic medical records, therefore those diagnosed with an outcome during a deployment were only classified as having an event if an additional diagnosis appeared in the medical record after the individual returned from deployment.

The majority of nondeployed Marines are unlikely to experience traumatic events resembling those of their deployed peers. It was not possible to assess which nondeployed Marines experienced traumatic events during the study period. Furthermore, nondeploying Marines may be fundamentally different from their peers since all Marines should be readily deployable; reasons for nondeployment were not possible to discern in this study. As experiencing a traumatic event is a necessary precursor to the development of PTSD, the population for this study included a subset of Marines who completed the RAP survey and were deployed in support of the conflicts in Iraq and Afghanistan between September 1, 2001 and June 30, 2004. Of the 9021 eligible responders, those who deployed prior to completing the RAP survey (n = 34), had a diagnosis of PTSD before or during their first deployment (n = 6), or had missing data (n = 590) were excluded from all analyses. When analyzing mental health disorders not specific to PTSD, responders were also excluded if they had a diagnosis for any of the mental health disorders prior to or during their first deployment (n = 167). Because MCRD San Diego does not train female Marines, analysis in this study was limited to men.

### Statistical analysis

Descriptive investigation of population characteristics was completed. Univariate analyses, including chi-square statistics and measures of association, were performed. Cox proportional hazard modeling was used to evaluate associations between ACE and PTSD. The 7 individual ACE categories were assessed together in 1 model, using a dichotomous "yes" or "no" response for each category. The degree of multiple ACE was examined in a separate model, which was categorized as those reporting no ACE, ACE in 1 category, and ACE in 2 or more categories. All multivariable modeling was adjusted for covariates that were significantly associated with the outcome or confounded the relationship between ACE and PTSD, all other variables were removed from the multivariable models. Follow-up time was calculated from the time of first deployment until (1) date of first event, (2) 2 years after the last deployment, (3) date of separation from the military, or (4) the end of the study, June 30, 2005, whichever occurred first.

All data analyses were completed using SAS Version 9.1 (SAS Institute, Inc., Cary, NC). This research has been conducted in compliance with all applicable federal regulations governing the protection of human subjects in research (NHRC Protocol 2000.0003). This study was approved by the Institutional Review Board of the Naval Health Research Center. Written consent was obtained from all study participants.

## Results

Of the 9021 RAP responders who were deployed in support of the current conflicts in Iraq and Afghanistan between September 1, 2001 and June 30, 2004, 8391 (93%) met the study criteria. Demographic characteristics of the entire study, and those diagnosed with postdeployment PTSD, are compared in Table 1. A greater proportion of those diagnosed with PTSD were white non-Hispanic, married, non-problematic drinkers, current smokers, deployed more than once, and diagnosed with a non-PTSD mental health disorder prior to their initial deployment. Additionally, a greater proportion of responders diagnosed with PTSD scored in the lowest quartile for AFQT, reported having 1 or fewer close friends, and reported poor mental health some, most, or all of the time at the beginning of military training.

**Table 1:** Characteristics of Recruit Assessment Program male responders deployed between September 1, 2001 and June 30, 2004

Characteristic	Study Sample N = 8391 (%)	Postdeployment PTSD^a^ N = 226 (%)
Age, y		
17-18	54.0	54.9
19-20	32.0	32.3
20+	14.1	12.8
Education		
High school or less	82.4	83.6
More than high school	17.6	16.4
AFQT score		
< 42 (below 25th percentile)	22.9	36.3
42-54 (25-49th percentile)	25.3	23.5
55-70 (50-74th percentile)	25.5	24.3
> 70 (above 74th percentile)	26.4	15.9
Parents' education		
High school or less	36.0	36.3
Some college	29.7	31.9
College graduate	27.8	25.7
Unknown	6.6	6.2
Race/ethnicity		
White non-Hispanic	63.8	68.1
Hispanic	23.6	19.9
Black non-Hispanic	4.5	4.0
Other	8.1	8.0
Marital status at time of deployment		
Not married	84.3	77.9
Married	15.8	22.1
Number of close friends		
0-1	12.1	16.8
2 or more	87.9	83.2
Alcohol drinking behavior at service entry		
Nondrinker	32.7	30.5
Non-problematic drinker	34.8	38.9
Potential problematic drinker	32.6	30.5
Smoking status at service entry		
Never	67.8	61.1
Past smoker	3.3	3.5
Current < 2 pack years	19.2	24.3
Current ≥ 2 pack years	9.7	11.1
Cumulative length of deployments^b^		
< 3 months	11.9	12.8
3-6 months	51.6	53.1
6-9 months	32.2	27.9
> 9 months	4.4	6.2
Number of deployments		
1	95.0	88.5
2-3	5.1	11.5
Diagnosed with mental health disorder prior to deployment		
No	98.1	95.6
Yes	1.9	4.4
Self-reported visiting mental health professional in last 5 years		
No	98.8	98.2
Yes	1.2	1.8
Self-reported poor mental health^c^		
None or a little of the time	78.0	74.8
Some, most, or all the time	22.0	25.2
Non-ACE past traumatic event^d^		
No	59.7	59.7
Yes	40.3	40.3

Abbreviations: ACE = adverse childhood experiences; AFQT = Armed Forces Qualification Test; PTSD = posttraumatic stress disorder.

As a result of rounding, percentages may not sum to 100%.

^a^Diagnosed with PTSD after returning from first deployment in support of the operations in Iraq and Afghanistan and within 2 years of last deployment. Responders with a PTSD diagnosis prior to first deployment are excluded from this model.

^b^Cumulative number of days deployed until first diagnosis, 2 years after last deployment, or June 30, 2005, whichever came first.

^c^Average of Likert scale responses to 4 survey questions asking about feeling downhearted and blue, nervous, down in the dumps, and not calm or peaceful, during the past year.

^d^Self-reported having a severe accident, seeing someone badly injured or killed, being attacked or assaulted, threatened with a weapon, or being raped.

Adverse childhood experiences of those diagnosed with PTSD were compared with the study sample (Table 2). A greater proportion of those diagnosed with PTSD reported childhood physical neglect, emotional neglect, emotional abuse, and domestic violence. Additionally, a greater proportion of those diagnosed with PTSD also self-reported a history of ACE in more than 1 category than the study sample in general.

**Table 2:** Adverse childhood experiences of Recruit Assessment Program male responders deployed between September 1, 2001 and June 30, 2004

Adverse Childhood Experiences	Study Sample N = 8391 %	Postdeployment PTSD^a^ N = 226 %
Physical neglect^b,c^		
No	88.5	81.0
Yes	11.0	19.0
Physical abuse		
No	96.8	96.0
Yes	3.0	3.1
Emotional neglect^b,c^		
No	93.3	88.9
Yes	6.2	10.6
Emotional abuse^c^		
No	92.4	89.4
Yes	7.1	10.2
Domestic violence		
No	92.2	89.8
Yes	7.5	9.7
Sexual abuse		
No	98.4	97.4
Yes	1.2	1.8
Family history of mental illness or alcohol abuse		
No	82.7	83.6
Yes	15.2	15.0
Overall ACE^c^		
None	66.5	61.1
ACE in 1 category	19.6	20.8
ACE in 2 or more categories	11.6	16.4

Abbreviations: ACE = adverse childhood experiences; PTSD = posttraumatic stress disorder.

As a result of missing data, percentages may not sum to 100%.

^a^Diagnosed with PTSD after returning from first deployment in support of the operations in Iraq and Afghanistan and within 2 years of last deployment. Responders with a PTSD diagnosis prior to first deployment are excluded from this model.

^b^Significant (*p *< .05) association with PTSD diagnosis.

^c^Significant (*p *< .05) association with mental health diagnosis.

Those who self-reported being physically neglected as a child were more likely to be diagnosed with postdeployment PTSD (hazard ratio [HR] = 1.74; 95% confidence interval [CI] = 1.17, 2.59), after adjusting for history of ACE in other categories, and other covariates and confounders (Table 3). Furthermore, Marines who were married at the time of deployment, deployed more than once, and reported being diagnosed with a mental health disorder were at greater risk for postdeployment PTSD, while those who scored anywhere above the 25th percentile on the AFQT were at a lower risk for postdeployment PTSD.

**Table 3:** Adjusted risk of posttraumatic stress disorder among deployed male Marines by adverse childhood experiences

	**Postdeployment PTSD**^**a**^	
	
Characteristic	Prevalence per 100	**HR (95% CI)**^**b**^
Physical neglect		
No	2.48	1.00
Yes	4.58	1.74 (1.17, 2.59)
Physical abuse		
No	2.70	1.00
Yes	2.88	0.58 (0.25, 1.32)
Emotional neglect		
No	2.57	1.00
Yes	4.80	1.30 (0.78, 2.15)
Emotional abuse		
No	2.61	1.00
Yes	3.97	1.30 (0.78, 2.16)
Domestic violence		
No	2.63	1.00
Yes	3.59	1.19 (0.75, 1.90)
Sexual abuse		
No	2.69	1.00
Yes	4.26	1.13 (0.40, 3.16)
Family history of mental illness or alcohol abuse		
No	2.72	1.00
Yes	2.61	0.88 (0.60, 1.29)
Marital status at time of deployment		
Not married	2.50	1.00
Married	3.83	1.48 (1.08, 2.04)
AFQT score		
< 42 (below 25th percentile)	4.29	1.00
42-54 (25-49th percentile)	2.54	0.60 (0.42, 0.85)
55-70 (50-74th percentile)	2.60	0.63 (0.44, 0.89)
> 70 (above 74th percentile)	1.62	0.40 (0.27, 0.60)
Number of deployments		
1	2.51	1.00
2-3	6.31	2.33 (1.54, 3.52)
Diagnosed with mental health disorder prior to deployment		
No	2.63	1.00
Yes	6.58	2.52 (1.33, 4.77)

Abbreviations: AFQT = Armed Forces Qualification Test; CI = confidence interval; HR = hazard ratio; PTSD = posttraumatic stress disorder.

^a^Diagnosed with PTSD after returning from first deployment in support of the operations in Iraq and Afghanistan and within 2 years of last deployment. Responders with a PTSD diagnosis prior to first deployment are excluded from this model.

^b^HR and 95% CI are adjusted for AFQT score, number of deployments, mental health diagnosis prior to deployment, marital status, and ACE categories.

After adjusting for covariates, those who reported ACE in 2 or more categories were at significant risk for being diagnosed with postdeployment PTSD (HR = 1.57; 95% CI = 1.09, 2.26) and mental health disorders not specific to PTSD (HR = 1.41; 95% CI = 1.10, 1.80) compared with those who reported no ACE (Table 4).

**Table 4:** Adjusted risk of posttraumatic stress disorder and mental health disorders among deployed male Marines by adverse childhood experiences

	**PTSD**^**a**^		**Mental Health Disorders not Specific to PTSD**^**b**^	
	
Adverse Childhood Experiences	Prevalence per 100	HR (95% CI)	Prevalence per 100	HR (95% CI)
History of ACE^d ^in multiple categories				
None	2.47	1.00	6.03	1.00
ACE in 1 category	2.86	1.21 (0.87, 1.69)	6.90	1.17 (0.94, 1.45)
ACE in 2 or more categories	3.81	1.57 (1.09, 2.26)	8.57	1.41 (1.10, 1.80)

Abbreviations: ACE = adverse childhood experiences; AFQT = Armed Forces Qualification Test; CI = confidence interval; HR = hazard ratio; PTSD = posttraumatic stress disorder.

^a^Models the risk of having a PTSD diagnosis after returning from first deployment in support of the operations in Iraq and Afghanistan and within 2 years of last deployment, after adjusting for AFQT score, number of deployments, mental health diagnosis prior to deployment, and marital status.

^b^Models the risk of a mental health diagnosis after returning from first deployment in support of the operations in Iraq and Afghanistan and within 2 years of last deployment, after adjusting for AFQT score, smoking status, race/ethnicity, and marital status.

## Discussion

Adverse childhood experiences, including neglect and abuse, have been associated with an increase in various diseases and negative health outcomes in adulthood, including depression, PTSD, alcohol-related problems, and even chronic fatigue syndrome in adulthood [13,15-17,20,21,26-29]. Previous studies have shown a graded relationship between the total number of ACE and negative health behaviors, including early onset of drinking alcohol and suicide attempts [17,20,21,30]. An association between ACE and alcohol problems has been demonstrated among the same population of young, male Marines examined in the current study [31]. Adverse childhood experiences, including sexual abuse, emotional abuse, physical abuse, and emotional and physical neglect, have been associated with combat-related PTSD in retrospective and cross-sectional studies [13-15,32,33].

The findings of this prospective evaluation found that Marines who reported ACE in 2 or more categories were at increased risk for PTSD after military deployment compared with those who reported no ACE exposure. The results of this report also indicate that men who reported physical neglect in childhood in particular were significantly more likely to be diagnosed with PTSD. Reports of other types of ACE, including sexual or physical abuse, emotional neglect or abuse, domestic violence or family history of mental health illness or alcohol abuse, were not independently associated with an increased risk for PTSD. Previous research has found no direct link between domestic violence, problematic family history, and PTSD [15,34]. Other ACE research, however, has reported associations between PTSD and physical and sexual abuse [14,15,32,33].

The concept that prior trauma, ACE or otherwise, confers increased vulnerability, rather than resilience, to post-combat PTSD is consistent with other recent, large, prospective studies in the US military. The Millennium Cohort identified the risk of post-combat PTSD as increased at least two-fold among a diverse group of military members, men and women, who survived prior adult trauma without evidence of PTSD, but subsequently deployed to a combat environment [35].

In a meta-analysis [8] of childhood adversity, younger age at time of exposure, less education, minority race/ethnicity, trauma severity, and lack of social support among veterans were identified as risk factors for developing PTSD. Consistent with previous research [36,37], men in this study who scored in the lowest quartile on an intelligence test (as measured by the AFQT) had an increased risk of being diagnosed with postdeployment PTSD. The findings of this study indicate that Marines with more than 1 military deployment were at increased risk for PTSD compared with their peers with only 1 deployment. While combat trauma data were not collected for this study, number of deployments may be related to trauma severity. In contrast to previous studies, younger age, less education, and minority race/ethnicity were not significantly associated with an increased risk for PTSD in this study [8]. The limited range in age and education among this study population may explain the lack of association with PTSD. Also in contrast to previous studies indicating lack of social support as a risk factor for PTSD [11,18,38,39], Marines in this study who were married at the time of deployment had a higher risk of being diagnosed with PTSD. It is possible that elevated rates of PTSD among married men are a result of spousal encouragement to seek medical care, and consequently be diagnosed with PTSD. And while the current study found the number of friends during recruit training not significantly associated with PTSD, it is reasonable that the number of friends recruits have in training may be different by the time they deploy.

Unlike many recent studies that have used self-reported screening tools for PTSD outcomes [17,18], this study used a standard diagnosis in electronic medical records to identify men with PTSD. Using outpatient and hospitalization records, 2.7% of Marines were diagnosed with postdeployment PTSD within 2 years following deployment. Previous studies using interviews and screening criteria indicate that PTSD among Marines and Army soldiers returning from the conflicts in Iraq and Afghanistan is as high as 4.7% to 19.9% [9,10]. Service members who screen positive for PTSD, however, may not subsequently be diagnosed with PTSD. Previous research suggests service members diagnosed with PTSD may represent a small subset of those who screen positive for PTSD using self-reported assessment tools. Hoge et al. [10] found that only 23% to 40% of service members who screened positive for a mental health disorder reported seeking mental health care. Negative stigma, a belief that seeking mental health care could harm one's career, and other barriers have been suggested as reasons that service members resist seeking mental health care [10]. Of those service members who accessed mental health services, approximately one third received a mental health diagnosis [9]. Therefore, men diagnosed with PTSD in this study may have been more willing to seek mental health care than their peers. Comparatively, it is possible that those willing to report ACE are also more likely to seek medical attention. As suggested, negative stigma may be one reason that service members do not seek mental healthcare and theoretically could also be a reason that the same service members are hesitant to report ACE.

### Strengths and limitations

This study has some limitations that should be considered. A study period of only 2 years may not be long enough to adequately capture all members who may be diagnosed with PTSD; although the onset of PTSD symptoms usually occurs within 3 months after a traumatic event [40]. Previous studies have identified combat exposures as one of the most consistent risk factors for developing combat-related PTSD [5,7,10,41]. While cumulative length of deployment was used as a covariate in this study, actual combat exposures could not be assessed in this study. These Marines may have been exposed to varying levels of combat or trauma, however, it is likely that the combat exposure experienced by this homogeneous sample of active duty, male Marines in this study was similar since they all deployed for the first time shortly after finishing recruit training [10]. Responders who had a previous PTSD diagnosis recorded in their military medical records or billed to the military were excluded, though it was not possible to account for PTSD diagnoses that may have occurred before responders joined the Marine Corps. Adverse childhood experiences were self-reported on a survey that was administered at the beginning of recruit training. Like all self-reported data, it is difficult to know the extent of recall bias and reporting bias, especially with a sensitive topic such as ACE. With the low prevalence of PTSD and the sample size of this study, there was insufficient power to detect small differences in the risk of being diagnosed with PTSD across exposure categories. We were able to identify significant differences in risk for more common exposures, such as physical neglect. Since this study was conducted among male Marines, the results may not be generalizable across all service branches or to female personnel.

Despite these limitations, this study had a number of strengths. Prospective design eliminated biases related to same-time assessment of exposures and outcomes. Medical records, an objective outcome measure, were used to assess PTSD in contrast to self-reported symptoms. The electronic medical records are very complete for active-duty service members since medical visits at Department of Defense facilities and those billed to the military are included in these records. Both hospitalization and outpatient data were used to assess PTSD diagnoses, allowing for the full spectrum of health effects to be examined. The all-male population provided unique perspective on ACE, which is not always well described among men. Proportional hazard modeling allowed for hazard risk estimation while adjusting for many covariates and varied length of follow-up.

## Conclusions

In summary, this study describes an at-risk population whose adverse childhood experiences appear to increase vulnerability to receiving a postdeployment PTSD diagnosis. Young, male Marine recruits who self-reported multiple types of ACE were at significantly increased risk for a postdeployment PTSD diagnosis. More specifically, those who reported childhood physical neglect were at greatest risk of being diagnosed with postdeployment PTSD. These findings suggest that reporting of ACE increases the risk of subsequent diagnosis of postdeployment PTSD among Marines. It is plausible that such a population could be targeted for PTSD prevention programs, early intervention after exposure to stress, or even protection from stressful exposures, when possible.

## Competing interests

The authors declare that they have no competing interests.

## Authors' contributions

CL and BS performed the statistical analysis. All authors helped conceive the study, participated in its design and coordination, and helped to draft the manuscript. All authors read and approved the final manuscript.

## Pre-publication history

The pre-publication history for this paper can be accessed here:

http://www.biomedcentral.com/1471-2458/10/437/prepub
